# Point-of-Care Abdominal Ultrasonography (POCUS) on the Way to the Right and Rapid Diagnosis

**DOI:** 10.3390/diagnostics12092052

**Published:** 2022-08-24

**Authors:** Tijana Radonjić, Maja Popović, Marija Zdravković, Igor Jovanović, Višeslav Popadić, Bogdan Crnokrak, Slobodan Klašnja, Olga Mandić, Marija Dukić, Marija Branković

**Affiliations:** 1 University Hospital Medical Center Bežanijska kosa, 11000 Belgrade, Serbia; 2 Faculty of Medicine, University of Belgrade, 11000 Belgrade, Serbia

**Keywords:** POCUS, point-of-care, abdomen, ultrasound

## Abstract

Point-of-Care ultrasound (POCUS) is based on target ultrasound that is performed wherever a patient is being treated, and by a non-radiologist directly involved in the patient’s care. It is used either for quick diagnosis or procedural guidance. Abdominal pain is one of the most common complaints in emergency departments, and POCUS can help in the differentiation of patients who need additional diagnostic tests or hospital treatment, which eventually reduces the overall costs of health care. POCUS has high sensitivity and specificity in abdominal pathology, it can be helpful in the evaluation of biliary, intestinal, and urinary tract, and it is especially used in trauma. Additionally, the gold standard for abdominal aortic aneurysm detection, follow up and screening is precisely this diagnostic procedure. Unfortunately, the quality of ultrasound examination can be affected by the experience of the physician performing it and the patient’s body weight. There is no doubt that POCUS is being increasingly recognized, but all motivated physicians should be provided with dedicated tutors and enough time for learning. This would certainly help to implement this diagnostic method as a routine in emergency and critical care departments, and significantly shorten the time until definitive diagnosis.

## 1. Introduction

Ultrasonography is very important in clinical practice as it is a low-cost, non-invasive, radiation-free diagnostic procedure that can be repeated multiple times with little risk to patients [1]. In particular, point-of-care ultrasound (POCUS) is even more significant as it is based on target ultrasound of various organs and it achieves a direct correlation with signs and symptoms [1,2]. POCUS is performed wherever a patient is being treated, but usually at the bedside, and by a clinician directly involved in the patient’s care [1]. This type of ultrasound examination answers a specific clinical question, and is an extension of the clinical examination that can cover different regions at the same time [3]. In other words, these two combined improve the accuracy of making a diagnosis and help a physician to decide about the next step in treatment [3]. Moreover, POCUS is performed at the point of care, either for quick diagnosis or procedural guidance, in real time [2]. Furthermore, it reduces the overall costs of health care, as the diagnosis can be made in a shorter period, avoiding tests that are not necessary at a given moment [4]. Nowadays, most ultrasound systems are portable, and even implemented in a laptop-sized housing or connected to a tablet and a cell phone, which eases its point-of-care use. 

In emergency departments, abdominal pain is the third most common indication of POCUS [5,6,7,8,9]. Acute abdomen is a sudden onset of abdominal pain that requires immediate medical attention [10], but is also a major diagnostic challenge with many differential diagnoses that can range from self-limiting illnesses to life-threatening conditions [11,12]. Patients with acute abdomen are usually referred for various tests [13], but half of these patients do not require hospital admission [14]. Consequently, POCUS, as one of the initial diagnostic procedures in these patients, can significantly contribute to a faster diagnosis, with a more rational use of existing resources, as well as to the adequate differentiation of patients who need additional diagnostic tests or hospital treatment [15]. In patients with acute abdominal pain, POCUS is most often used to evaluate the biliary, intestinal, and urinary tract, and aneurysm of the abdominal aorta, but it also has a crucial role in the evaluation of patients who have had trauma [16,17].

Additionally, POCUS is clinically important in shock status assessment as it provides the examination of different organs systematically at the same time [18]. Shock is an emergency condition, and this diagnostic procedure can help a physician to make a critical decision on the spot and very quickly [18]. This is why POCUS is valuable in emergency departments, intensive care units, and operating rooms [18], and it is becoming a routine approach to the care of critically ill patients [19,20]. This role, along with procedural guidance, implies that POCUS is experiencing a golden age in critical care and emergency medicine and this is increasingly being recognized [21,22].

## 2. Intraperitoneal Free Fluid

**Figure 1:**
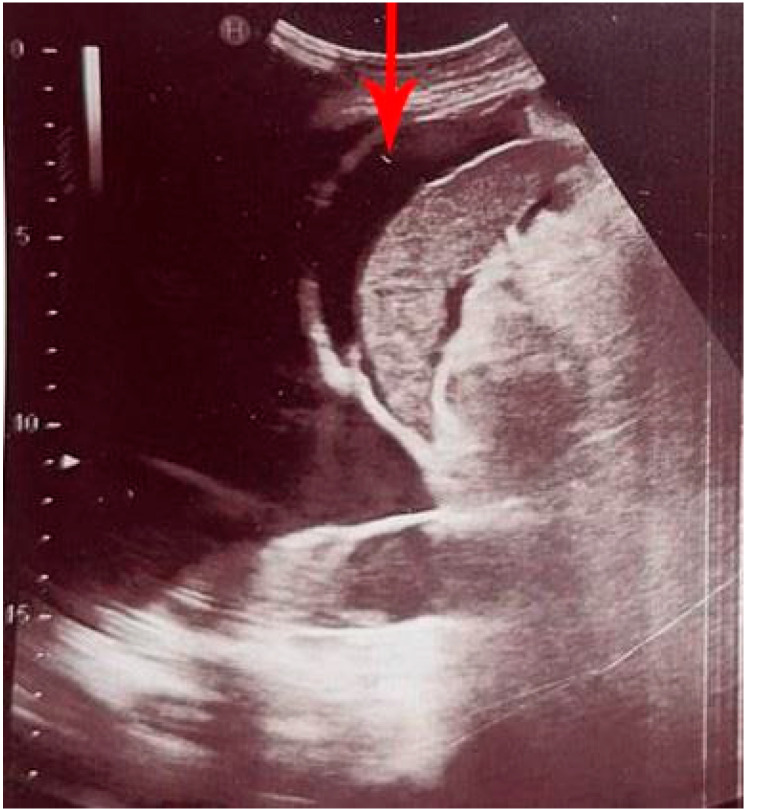
Peri-splenic intraperitoneal free fluid (IPF) (red arrow).

Intraperitoneal free fluid (IPF) is a pathological accumulation of fluid in the peritoneal cavity, which occurs as a symptom of various diseases [22]. The most common causes of IPF in emergency patients are traumatic hemorrhage, ascites, bile, urine leak, and ruptured ectopic pregnancy [23]. The initial diagnostic procedure used to detect and quantify the amount of free fluid is abdominal ultrasound (US), which can also help to determine the cause of IPF accumulation. Additionally, abdominal US can offer guidance during paracentesis, to accurately determine the localization of the IPF, but also to ensure that the spleen and other important structures are not injured during the procedure [24,25]. US has a sensitivity and specificity in the detection of IPF of over 90%, while up to 10 mL of free intraperitoneal fluid can be detected by POCUS in experienced hands [26,27]. In the abdomen, IPF is most often localized perihepatic (in Morrison’s pouch) or peris-plenic (Figure 1), while in the pelvis it is most often localized in the Douglas pouch [28]. 

Based on the amount of IPF present, the classification is made into three grades: a small amount (grade 1), which can only be detected by ultrasound, a moderate amount (grade 2), which manifests as moderately symmetrical abdominal distension, and a large volume (grade 3) with pronounced abdominal distension [24]. On US examination, the fluid is usually anechoic (black), while clotted blood can be gray and difficult to identify [23], which is precisely one of the critical challenges with POCUS, i.e., the inability to define the type of fluid (urine, bile, blood or ascites) [23]. Despite this shortcoming, the detection of IPF by ultrasound is crucial in trauma management, as also in the detection and characterization of ascites [29]. 

Bedside ultrasound can also be used during paracentesis [21], which can be effective in eliminating the need for decompressive laparotomy in abdominal compartment syndrome [30]. The most commonly used approach for paracentesis is through the left lower quadrant, 3 cm cranial and 3 cm medial to the anterior superior iliac spine [23]. In addition to this approach, access through the right lower quadrant and the midline linea alba between the umbilicus and pubic bone can be used [31]. It is recommended that the procedure should be performed under sterile conditions [23]. Complications rarely occur, but the most common are hematoma of the abdominal wall (1%), hemoperitoneum (<0.1%), intestinal perforation (<0.1%) and infection (<0.1%) [31,32].

## 3. Focused Assessment with Sonography in Trauma (FAST)

**Figure 2:**
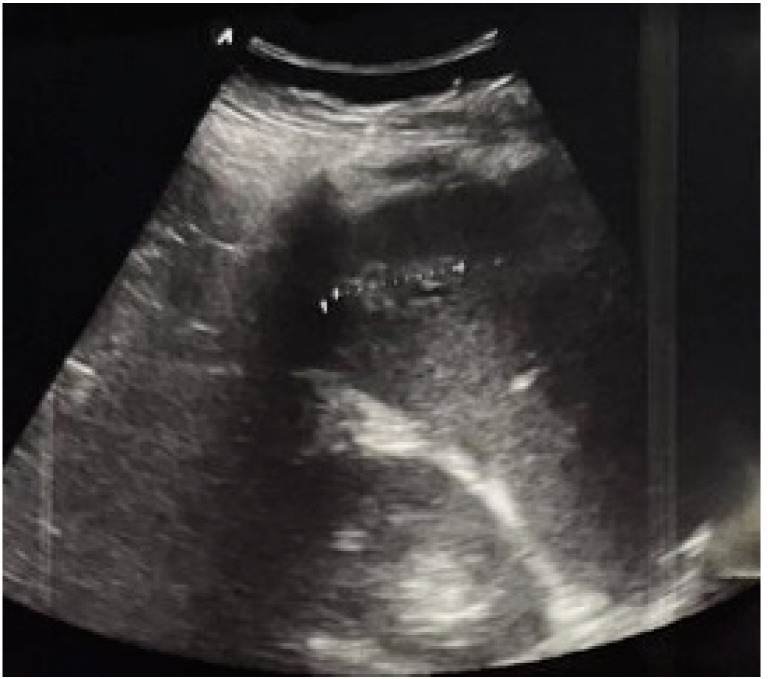
Splenic trauma (diameter of hyperechogenic focus 31.2 mm).

Besides the physical examination, in trauma Focused Assessment with Sonography in Trauma (FAST) is also clinically very important to assess for hemoperitoneum, while it provides an additional noninvasive examination of the abdomen with minimal disruption of resuscitation efforts [33]. In addition, it can be performed at the site of trauma or during transportation of the patient. It has a high specificity (99%) for diagnosing free fluid, but it also has a low sensitivity (60–80%), so free fluid cannot be ruled out [34]. In trauma patients, FAST can be performed quickly and if it is initially negative, it can be repeated if needed, according to the clinical status of the patient. On the other hand, if it is positive, and with the presence of hemorrhagic shock, it indicates intraabdominal bleeding and the need for procedural or operative treatment [35]. The main issue is the amount of blood that can be seen using US, which gives an advantage to computed tomography (CT) scan examination [36]. This can explain why 27–29% of patients with splenic injury verified by CT scan examination, did not have US detectable hemoperitoneum, and why this injury can be easily missed [33]. It should be emphasized that, in trauma, solid organ injury could be assessed with FAST (Figure 2), but only if it is not time consuming and doesn’t interfere with resuscitation. Unfortunately, in this case, the examination has low sensitivity (41–44%), as freshly coagulated blood has a similar echogenicity to the parenchyma of the injured solid organ [34].

### 3.1. Extended Focused Assessment with Sonography in Trauma (E-FAST)

There is another type of Focused Assessment with Sonography in Trauma called the extended FAST (E-FAST) which includes pneumothorax and hemothorax assessment [37]. This diagnostic procedure has higher sensitivity and specificity than chest radiography [38,39], and the evidence shows that half of pneumo-thoraces are missed on an X-ray [40]. Pneumothorax is one of the most common serious thoracic injuries that can lead to lethal outcome, but is preventable [41], and this is why E-FAST is very useful in everyday practice in emergency departments. In regard to hemothorax, in contrast to chest radiography, E-FAST can even detect 20 mL of fluid in the pleural space [42]. Unfortunately, traumatic aortic pathology cannot be assessed using this diagnostic procedure, so in this case CT angiography must be used [43].

### 3.2. Pneumoperitoneum

The presence of air in the peritoneal cavity, pneumoperitoneum, is often caused by perforation of a hollow abdominal organ as a result of cholecystitis, diverticulitis, appendicitis, trauma, bowel malignancy or ischemia [44]. When diagnosing pneumoperitoneum, the evidence shows that POCUS is again superior to X-ray examination, although CT scan is the gold standard [45]. Despite this, US does not create unnecessary radiation exposure, which is a huge advantage over the other two diagnostic methods. Though, there are suggestions that pneumoperitoneum assessment should be implemented in FAST protocol, the longer duration of the procedure is certainly an issue, so the recommendation is to extend the examination only if it does not interfere with resuscitation [46].

### 3.3. Ectopic Pregnancy

An ectopic pregnancy is a potentially life-threatening condition that is frequently seen in gynecology emergency departments. It is the presence of a fertilized embryo that implants outside of the uterus, most commonly in the fallopian tube [47]. Fallopian tube rupture due to ectopic pregnancy results in intraabdominal hemorrhage, and this is also the case when making an accurate diagnosis in good time prevents lethal outcome. Here, FAST should be an additional part of the US examination as it could reveal hemoperitoneum, while POCUS is already broadly used in gynecology due to its high specificity for the detection of intrauterine pregnancy [48]. In gynecology, FAST scan includes views of the right upper quadrant, and the suprapubic view (pouch of Douglas) [48]. The evidence shows that moderate to large free fluid found in the pelvis highly indicates ruptured ectopic pregnancy with a specificity of 94%, while the presence of free fluid in the right upper quadrant has a specificity of 99.5% for the same diagnosis [48].

## 4. Cholelithiasis and Cholecystitis

Cholelithiasis is the presence of gallstones in the gallbladder [49], which occurs in 10% to 15% of the adult population and is asymptomatic in most cases [50]. In addition, it can also be symptomatic, when it is most often manifested by inflammation of the gallbladder (cholecystitis), due to obstruction of the cystic duct by a gallstone [49]. In 95% of cases, acute cholecystitis is caused by cholelithiasis, while in the remaining cases it is acalculous cholecystitis [51,52]. Moreover, gallstones can also be found in the lumen of the common bile duct which leads to choledocholithiasis. This condition can be complicated by inflammation of the bile duct (cholangitis) [49], which is why the diameter of the bile duct should always be measured during an US examination, as its dilatation indicates obstruction [3].

The most common manifestation of acute cholecystitis is biliary colic, defined as the sudden onset of pain in the epigastric region or the right upper quadrant of the stomach. Biliary colic peaks within one hour of the onset [50] and then the pain gradually weakens, lasting up to 5 to 6 h [49]. Acute cholecystitis or cholangitis should be suspected in patients with persistent pain, lasting more than 5 h, associated with fever and/or elevated values of inflammatory markers [49]. Symptoms of acute cholangitis are characterized by Charcot’s triad which includes fever, jaundice, and abdominal pain. The most common complication of cholecystitis and cholangitis is pancreatitis [49].

**Figure 3:**
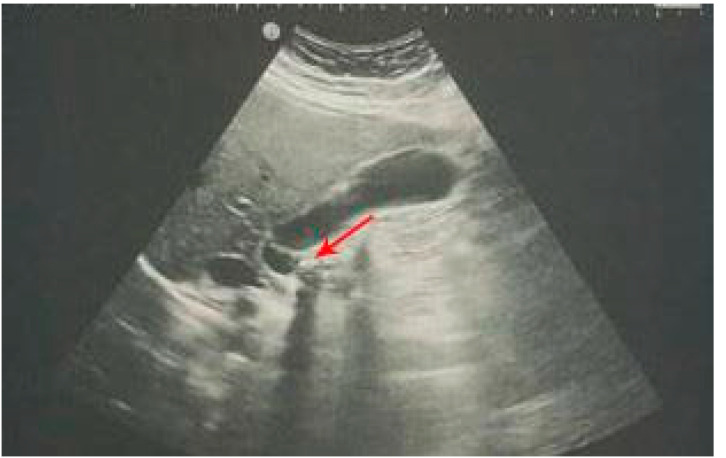
An impacted calculus in the neck of the gallbladder (red arrow).

The gold standard for the diagnosis of cholelithiasis (Figure 3) is an US examination [53] which is especially important for pregnant women, as this procedure does not use ionizing radiation [49]. Furthermore, studies have shown that POCUS has great sensitivity (89.8%) and specificity (88%) for the detection of cholelithiasis [54], but can also detect biliary sludge, which is the initial stage of gallstones formation [49].

A gallstone less than 5 mm in diameter presents as a hyperechoic focus without a posterior acoustic shadow [55], whereas a gallstone larger than 5 mm in diameter presents as a hyperechoic focus with a posterior acoustic shadow and an echogenic rim [3]. Signs of acute cholecystitis on US examination are an enlarged gallbladder, thickened (thickness greater than 4 mm) and stratified walls, biliary sludge and gallstones in the lumen, pericholecystic fluid and a positive Murphy’s sign (painful sensitivity under the right costal arch to probe pressure) [16,56,57]. Detecting gallstones during POCUS associated with positive Murphy’s sign has a 92% positive predictive value for acute cholecystitis [51]. If the US examination shows a physiological gallbladder wall and a physiological common bile duct, acute cholecystitis can be ruled out [49].

The results of the study which comprised 1690 patients with abdominal pain showed that POCUS had 88% sensitivity, 87% specificity, 91% positive predictive value, and 83% negative predictive value in the diagnosis of gallstones [58]. Moreover, in patients with a negative POCUS, surgery or admission for cholecystitis within two weeks of the study was unlikely [59].

## 5. Urolithiasis and Hydronephrosis

Renal colic most often occurs as a result of urolithiasis, i.e., the presence of calculus in the urinary tract [3]. It manifests with an acute onset of cramping, severe unilateral pain in the abdomen, and flanks, which can migrate along the projection of the ureter, following the movement of the kidney stone down the ureter, on its way from the kidney to the bladder [60,61]. The pain occurs in episodes, each usually lasting 20 to 60 min, and not stopping completely until the next episode [61]. The most common symptoms of urolithiasis, including pain, are malaise, sweating, nausea and vomiting, but also fever and chills [62]. Statistically, between 3% and 15% of adults experience renal colic during their lifetime, and it is more common in males between the ages of 20 and 50 [63]. In patients with suspected renal colic, the US examination should be done to confirm/exclude the presence of urinary tract obstruction and to confirm/exclude the presence of calculus. Additionally, if calculus is present, the information on the size and location of the calculus should be provided, as well as all detected complications. On the other hand, all potential disorders mimicking renal colic must be excluded during the US examination [61]. Unfortunately, in the detection of calculi, POCUS has low sensitivity, i.e., it can detect calculi proximal to the ureteropelvic junction or distal to the ureterovesical junction where there is an acoustic window, while it is very difficult to visualize the retroperitoneal ureter between the kidney and the bladder [3]. On the other hand, POCUS can reveal a dilated ureter, which, associated with acute abdominal pain, indicates distal obstruction [3]. In conclusion, in renal colic, the main goal of POCUS is to confirm/exclude obstructive uropathy [64].

**Figure 4:**
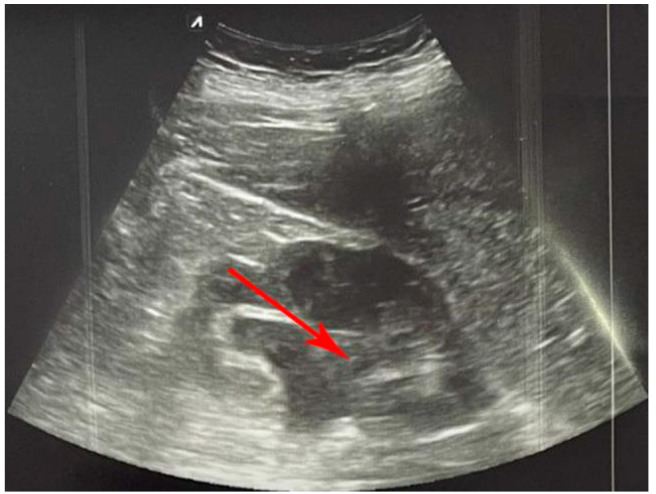
Severe hydronephrosis (left kidney) (red arrow).

Urolithiasis often can lead to hydronephrosis, which is a frequent but reversible cause of acute kidney injury [65]. Hydronephrosis occurs more often in patients with stones greater than 5 mm in diameter compared to patients with stones less than 5 mm in diameter [66,67]. Moreover, it can occur due to internal obstruction of the ureter (e.g., a mass in the ureter) or due to external compression of the ureter by an abdominal aortic aneurysm, advanced pregnancy or pelvic mass, but also due to distal obstruction (e.g., prostate hypertrophy), when bilateral hydronephrosis occurs, so it is important to carry out the US examination on both kidneys [68]. With POCUS examination, hydronephrosis can be detected with high sensitivity and specificity, and additionally the degree of hydronephrosis (Figure 4) can be determined [65]. Depending on the body constitution of the patient and the experience of the physician, the sensitivity of POCUS is 72% to 97%, while the specificity is 73% to 98% [64,69]. Interestingly, in one study, it was found that sensitivity and specificity are greater than 90% for detecting hydronephrosis using POCUS performed by internal medicine physicians who had 5 h of training, compared with traditional ultrasonography [70].

POCUS is also used for evaluation of the bladder, allowing the differentiation of a distended bladder (a large fluid-filled structure) from ascites, assessment of bladder volume, and visualization of a Foley balloon within the bladder (confirmation of an adequately placed urinary catheter) [68].

## 6. Abdominal Aortic Aneurysm

An abdominal aortic aneurysm (AAA) is a permanent and irreversible, localized, enlargement of the lumen of the abdominal aorta [71]. Dilatation of the lumen may involve any segment of the abdominal aorta, most commonly the infrarenal segment (distal to the renal arteries) [71], and in this segment the diameter is over 30 mm [72]. In clinical practice, it is important to emphasize that small AAA is not considered for repair (<55 mm), but large AAA (≥55 mm) should be considered for surgical repair [73].

Recently, there has been an increasing trend in the incidence of AAA, due to the aging of the population, the increase in the number of smokers, but also the introduction of screening programs, and the improvement of diagnostic tools [71]. Among the population older than 50 years, AAA is detected in 2% to 5% of the population [74], and it occurs more often in men, more precisely between 1.3% and 8.9% in men and between 1% and 2.2% in women [75,76,77,78,79]. In most patients, aneurysms are asymptomatic and are most often discovered accidentally or due complications [80]. AAA can mimic other pathological conditions such as renal colic or acute diverticulitis, resulting in its later detection [74]. The most serious complication of AAA is rupture, which is a life-threatening emergency with high mortality [81]. Rupture is followed by a triad of symptoms: acute, severe pain in the central part of the abdomen or flank (which can radiate to the groin), shock and the presence of a pulsating abdominal mass [71]. The most important risk factor for aneurysm rupture is its size [82], so the recommendation is that patients with large aneurysms should undergo surgical intervention [82]. What is crucial is early screening [3] as it reduces ruptured AAA mortality by 34% [83]. One-time screening is recommended in men between 65 and 75 years of age, in smokers, especially those who have smoked at least 100 cigarettes in their lifetime, and in patients with comorbidities such as arterial hypertension, peripheral vascular disease, dyslipidemia or diabetes mellitus [84,85]. Interestingly, in patients with cardiovascular risk, with POCUS examination 71% of AAA can be diagnosed [84,85].

The gold standard for AAA detection, follow up and screening is US examination, as it is characterized by a sensitivity of 94% to 99% and a specificity of 98% to 100% [86,87,88,89,90]. US examination should comprise scanning of the aorta from the epigastrium to the distal bifurcation, including differentiation of its shape and caliber. More precisely, the outside diameter should be measured, and any thrombus in the lumen or dissection sign should be noted [3,35]. In addition, Color Doppler (CD) US plays an important role during the examination, showing the flow within an aneurysm [3]. Furthermore, CD US has a high specificity for aortic dissection, which is why it is an important tool for visualization of the intimal dissection flap, if any [91]. Using POCUS in primary care resulted in 100% sensitivity and specificity for AAA screening, two studies showed [84,89]. In one AAA screening study in which US examination was used, 1010 male patients were included, with a mean age of 71.3. Their results revealed that the median aortic diameter of included patients at diagnosis was 35 mm, but 47 mm in patients who were diagnosed with AAA incidentally [92]. This is why the use of POCUS for AAA screening in the primary care setting is strongly recommended and supported [92]. In the end, it must be highlighted that AAA screening can be difficult in obese patients [3].

## 7. Intestinal Obstruction

Bowel obstruction is a frequent gastrointestinal emergency, that requires prompt and effective treatment [18]. Obstruction means partial or complete interruption of the passage of solid, liquid and gaseous material inside the intestinal lumen [93]. Small bowel obstruction (SBO) or large bowel obstruction (LBO) is distinguished depending on the part of the intestine where the disruption of the passage occurred [93]. SBO can be proximal (high SBO) or distal (low SBO) [94]. SBO occurs more often, in about 60–85% of obstructions, compared to colonic obstruction, which includes 10–15% of obstructions [95]. In emergency departments, 2–8% of patients are patients with SBO, and of these patients about 16% are admitted to the surgical unit [96,97,98]. The most common cause of SBO is adhesion, in 55–75% of cases [99], followed by hernias and tumors [100,101,102]. Tumor is the cause of colon obstruction in about 60% of cases, while volvulus and diverticular disease occur in about 30% [94,103]. In the case of intestinal obstructions, the risk of complications is high, and strangulation occurs in 30% of patients, while intestinal necrosis occurs in 15% [93]. Both of these conditions lead to further progression to perforation, sepsis, and death [93]. Elderly patients, patients with comorbidities and patients who are diagnosed with delay have an increased risk of intestinal obstruction complications, which is why a rapid diagnosis is necessary [93,97,104].

Diagnosis involves imaging methods, as neither the presence nor absence of clinical features or isolated laboratory findings are sufficient criteria to exclude or confirm strangulation or intestinal necrosis [93]. In such cases, with acute abdominal pain and signs or symptoms of bowel obstruction, POCUS can be used as a supplement to the physical examination for rapid differentiation of the cause of the pain, as well as the assessment of the need for further diagnostics [99]. This diagnostic procedure can confirm or rule out intestinal obstruction, but can also indicate signs of other conditions such as the thickened wall in the case of inflammatory disease (appendicitis, diverticulitis or inflammatory bowel disease) or the existence of a “pseudo-kidney” in the case of a tumor [99]. When bowel obstruction is suspected, POCUS has 90% sensitivity and 96% specificity [105]. Compared to X-ray, US examination has the same sensitivity (less than 70%), but is more specific than X-ray [99]. Using POCUS, if there is obstruction, whether the obstacle is mechanical or functional, and its location can all be verified, along with if there is intestinal ischemia or necrosis. Additionally, using this diagnostic procedure, it is possible to follow up on the clinical progress of the patient treated conservatively [106]. Ultrasonography signs that indicate SBO are dilated bowel (>25 mm), thickened intestinal wall (>3 mm), length of the affected segment (>100 mm), bowel with content, accelerated, slowed down or absent peristalsis, enlarged and visible valvulae conniventes (>2 mm), as well as a collapsed lumen of the colon [106,107,108]. If the US examination detects enlarged loops of the small intestine, full of gas and liquid, without peristalsis, associated with gases of the large intestine, liquid, or feces, the finding is highly suspicious of paralytic ileus [18].

POCUS can detect the location and potential cause of obstruction in the small intestine, by analyzing the zone where the transition from dilated to collapsed bowel occurs, based on the visibility of valvulae conniventes, which become prominent in jejunal obstruction and are absent or rare in obstruction of the ileum [18,109]. It is also important to look for the cause of obstruction at the transition point between expanded proximal and collapsed distal loops [18]. Using US examination, it is possible to diagnose several pathologies including hernias, intussusception, ascariasis, foreign bodies, and tumors [110]. When the US examination reveals dilatated bowel, filled with liquid, with a thickened wall (>3 mm), absent peristalsis, and free fluid between intestinal loops, the finding is highly suspicious for ischemia [109]. When ischemia occurs, there is a reduction or absence of peristalsis, even in the case of mechanical obstructions [18]. The absence of peristalsis is considered as the absence of a peristaltic wave for longer than five minutes [111]. The use of CD US also plays an important role in the diagnosis of ischemia, as it gives information about the perfusion of the intestinal wall [112]. US signs of intestinal obstruction (thickened intestinal wall, absence of peristalsis, and presence of free fluid) together with clinical data of the patient are crucial for deciding on early surgical intervention [18]. Unfortunately, the quality of the US examination can be reduced due to obesity and meteorism, but also it can be affected if the physician performing it is not sufficiently experienced [99,105].

## 8. Diverticulitis

Diverticula are the most common changes in the colon [113]. These are pouch-like protrusions, precisely mucosal and submucosal hernias in the wall of the colon [113,114]. Diverticulosis occurs in about 60% of patients over the age of 60 [115,116], while the prevalence in people under the age of 40 is low [116]. Most often, diverticulosis is asymptomatic [117], but 15% to 25% of patients develop diverticulitis [118], which is usually the cause of abdominal pain [3], as well as the most common cause of non-traumatic colon perforation and elective colon resection [119,120]. Diverticulitis can occur in any part of the large intestine, most commonly in the sigmoid colon [121,122,123], which is why in females both transvaginal and transabdominal US examinations are useful [124]. On the other hand, it can occur in the small intestine as Meckel’s diverticulitis [122,123]. In about 85% of patients it manifests itself as a mild form (acute uncomplicated diverticulitis), in the form of peri-diverticular inflammation limited to the colon wall, but also as a severe form (complicated diverticulitis) [120,121]. Complications mainly include perforation with consequent peritonitis and hemoperitoneum [113], then abscess, fistula, or stricture [125,126], with abscess being the most common [125].

The most common clinical manifestation of acute diverticulitis is the triad consisting of severe abdominal pain, fever and elevated inflammatory markers [127,128]. Rectal bleeding, urinary symptoms [3], as well as changes in bowel habits, nausea, and vomiting can also occur [122]. The manifestation is also influenced by the localization of the inflammatory process, so when the right colon is involved it can mimic appendicitis [123].

As already known in clinical practice, the accuracy of physical examination for diverticulitis is low [3]. In this case, POCUS can help and lead to a diagnosis [3]. This diagnostic procedure is the first choice for diagnosing diverticulitis, which was the conclusion of one systematic review [3]. Additionally, the graded compression technique of ultrasound in diagnosing diverticulitis was the subject of another systematic review in which a sensitivity of 90% and specificity of 89% were reported [3]. Despite CT examination being thought to be more accurate than US in diagnosing diverticulitis, the conclusion of one systematic review with meta-analysis does not support that. It is shown that US and CT examinations have almost the same sensitivity (92% and 94%, respectively) and specificity (90% and 99%, respectively) [3,113].

Ultrasonography signs of diverticulitis include hypoechoic mural thickening of the colon wall (>4 mm), non-compressible target sign, and absence of peristalsis [18,129,130]. A segment of the colon affected by inflammation may have a rounded, folded edge, giving it the classical saw-tooth pattern [18]. Unlike malignant tumors, in diverticulitis the layers of the colon wall are usually preserved [129]. As a consequence of the inflammation, there is also a reaction of the surrounding fatty tissue, which is shown as echogenic on US examination [130]. In addition, POCUS can also detect complications of diverticulitis, such as a formed abscess, free intraperitoneal fluid, and free intraperitoneal air [131,132,133].

## 9. Appendicitis

**Figure 5:**
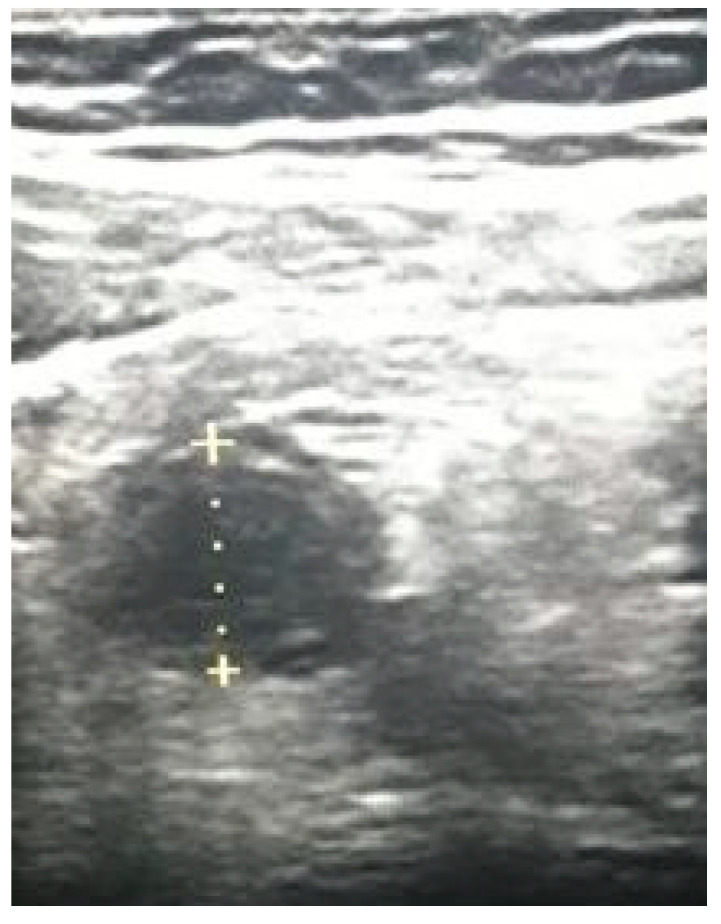
Acute appendicitis (diameter of appendix 11.9 mm).

Appendicitis, as the most common surgical emergency, occurs at a rate of 96.5 to 100 patients per 100,000 adults per year [134], with a peak between the age of 10 and 30, and it is less frequently seen in both extremes of age [135]. Appendicitis occurs as a result of obstruction of the appendix lumen, most often by coprolith, lymphoid hyperplasia or impacted stool, while the less commonly seen cause is a tumor of the appendix or cecum [135]. Lumen obstruction leads to inflammation, ischemia, necrosis, and possible subsequent perforation of the appendix [3]. Most often, the initial symptoms are periumbilical colic pain around the midgut, clustered localized pain that coincides with parietal irritation of the peritoneum [136] and within 24 h the pain intensifies with accompanying nausea, vomiting, and loss of appetite [137]. Acute appendicitis (Figure 5) is the most common cause of acute abdomen in younger patients [138].

**Figure 6:**
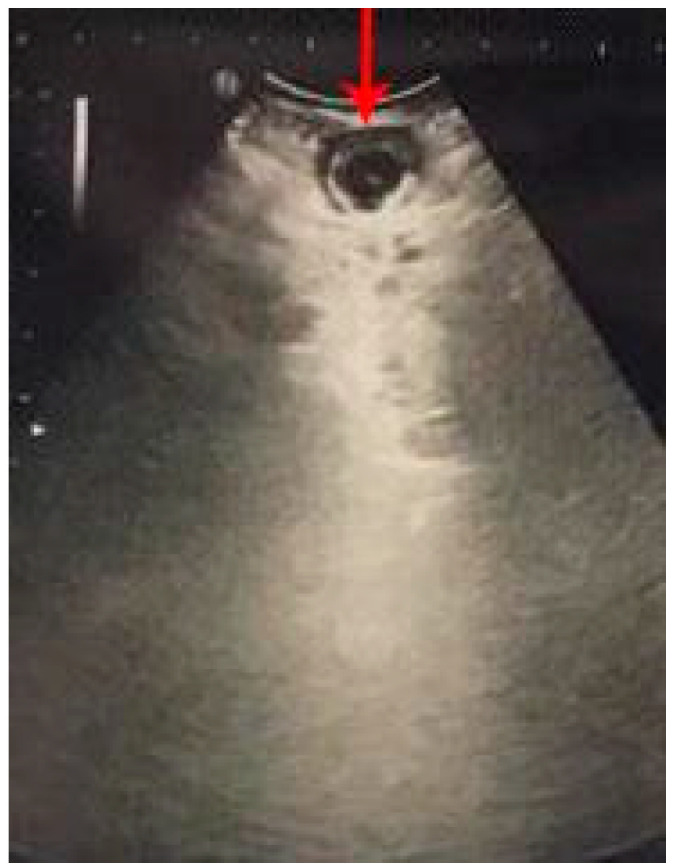
Target sign (red arrow).

The diagnosis of appendicitis is challenging and requires a synthesis of clinical, laboratory and radiological findings [138]. If the diagnosis is not made in time, perforation and significant morbidity can occur [139]. Nowadays, the evidence suggests that perforation is not necessarily an inevitable result of appendiceal obstruction, and even that resolution may be a common occurrence [138]. Multiple imaging modalities play an important role in the diagnosis of acute appendicitis [18], whereby ultrasound is the first modality of choice for all age groups, especially in children and pregnant women, due to its safety [139,140,141]. POCUS, when performed by surgeons and physicians working in the emergency department, has an overall sensitivity of 91% for appendicitis [139]. A meta-analysis showed that POCUS, when performed by emergency physicians, has 84% sensitivity and 91% specificity, with higher accuracy in children [142]. The physiological diameter of the appendix is between 4.4 and 5.1 mm [143], while a diameter greater than 6 mm indicates acute appendicitis when it is accompanied by appropriate clinical findings [18]. Trout et al. showed that an appendix diameter of 6 to 8 mm and more than 8 mm has the highest accuracy in the diagnosis of appendicitis (65%, 96%, respectively), but if the appendix diameter is less than 6 mm, appendicitis is diagnosed in only 2.6% of cases [144]. Direct ultrasound signs of appendicitis are a noncompressible tubular structure with a target sign greater than 6 mm in diameter at the site of the appendix (Figure 6), appendicolith, and hypervascularity on Doppler ultrasound [18]. In addition to these signs, free fluid around the appendix, abscess formation, increased echogenicity of mesenteric fat, enlarged local mesenteric lymph nodes and thickened wall can be detected [18,140].

The main disadvantage of POCUS is that the quality of the examination is affected by the patient’s body weight, but other disadvantages include the experience of the physician, pain during the procedure and intestinal gases [35]. Despite these shortcomings, POCUS is advised as the first diagnostic modality for acute appendicitis [139,140], and when the appendix is visualized, POCUS has a diagnostic sensitivity of almost 100% and a specificity of 85% [145]. On the other hand, CT is a more accurate diagnostic procedure in acute appendicitis [146], but it is a method that uses ionizing radiation and carries an unnecessary risk primarily for children, younger patients, and pregnant women. This is why POCUS, the leading diagnostic method, has advantage over CT examination [138].

## 10. Conclusion

The evidence shows that POCUS is certainly the best additional examination method, which leads to a faster and more accurate diagnosis. It can be performed wherever a patient is being treated and by a non-radiologist, which are the most important advantages of this diagnostic procedure. We are lucky nowadays to have US machines available in almost every medical institution, and it was shown that non-radiologist physicians can be trained quickly. Despite possible rapid learning, the quality of the examination can be affected by the experience of the physician. Usually, the patient’s body weight also influences the quality, which is another common disadvantage of this procedure. The mission is to provide dedicated tutors and enough time for learning to all motivated physicians. In summary, POCUS should be implemented as a routine procedure in clinical practice, especially in emergency and critical care departments, as it can provide less time consumption and establish new standards of care.

## Data Availability

Not applicable.
